# Atypical Presentation of Herpes Zoster Duplex *Bilateralis* in a Renal Transplanted Patient

**DOI:** 10.3390/healthcare2010020

**Published:** 2013-12-20

**Authors:** Ana Isabel Gouveia, João Borges-Costa, Luís Soares-Almeida, Alice Santana, José Guerra

**Affiliations:** 1 Universitary Clinic of Dermatology, Santa Maria Hospital, Avenue Prof. Egas Moniz, Lisboa 1649-028, Portugal; E-Mails: anaisabelgouveia@hotmail.com (A.I.G.); soaresdealmeida@hsm.min-saude.pt (L.S.-A.); 2 Dermatology Research Unit, Medicine Faculty, Lisboa University, Avenue Prof. Egas Moniz, Lisboa 1649-028, Portugal; 3 Microbiology and Tropical Clinic Units, Higiene e Medicina Tropical Institute, Lisboa New University, Lisboa 1349-008, Portugal; 4 Nefrology Unit, Santa Maria Hospital, Avenue Prof. Egas Moniz, Lisboa 1649-028, Portugal; E-Mails: alicesantana1@sapo.pt (A.S.); joseoliveiraguerra@gmail.com (J.G.)

**Keywords:** herpes zoster, immunosuppression, infection skin disorders in organ transplant recipients

## Abstract

Viral infections in renal transplant patients are an important cause of morbidity and mortality. In most cases, the clinical presentation of herpes zoster allows the diagnosis to be made only by history and physical examination. However, patients who are immunosuppressed may have uncommon presentations, and require a high index of suspicion and additional diagnostic testing for proper management. We report a rare presentation of herpes zoster *duplex bilateralis* involving symmetrical dermatomes over the lower limbs occurring in a woman with a recent history of renal transplantation. The skin lesions were also atypical representing a diagnostic challenge. This infection should be part of differential diagnosis of cutaneous manifestations in organ transplant recipients.

## 1. Introduction

Viral infections in renal transplant (RT) patients are an important cause of morbidity and mortality [1]. Immunosuppressive drugs are essential for organ and patient survival; however, they produce important inhibitory effects on immune defense mechanisms favoring the occurrence of complications, particularly infections and malignancies [2].

Varicella-zoster virus (VZV) is a member of the alpha subgroup of herpes viruses and cause two distinct clinical diseases, depending on whether infection is primary (varicella or chickenpox) or reactivation of latent VZV (herpes zoster or shingles) [3]. Primary VZV in the solid-organ transplant (SOT) recipients is rare. It is more frequent among the pediatric transplant population and can cause a life-threatening disseminated infection [3]. Approximately 3% of patients on a waitinglist for renal transplantation are seronegative for VZV and, therefore, are at risk to develop primary VZV infection after transplantation [4].

Herpes zoster (HZ) occurs in up to 13% of renal transplant patients [5]. Immunosuppressed patients with this infection may have atypical presentations, more severe complications and a greater tendency for prolonged course of disease [6]. Rarely, noncontiguous dermatomes are involved and in this case are named zoster *duplex unilateralis* or *bilateralis*, depending whether one or both halves of the body are involved [7].

## 2. Results and Discussion

**Figure 1:**
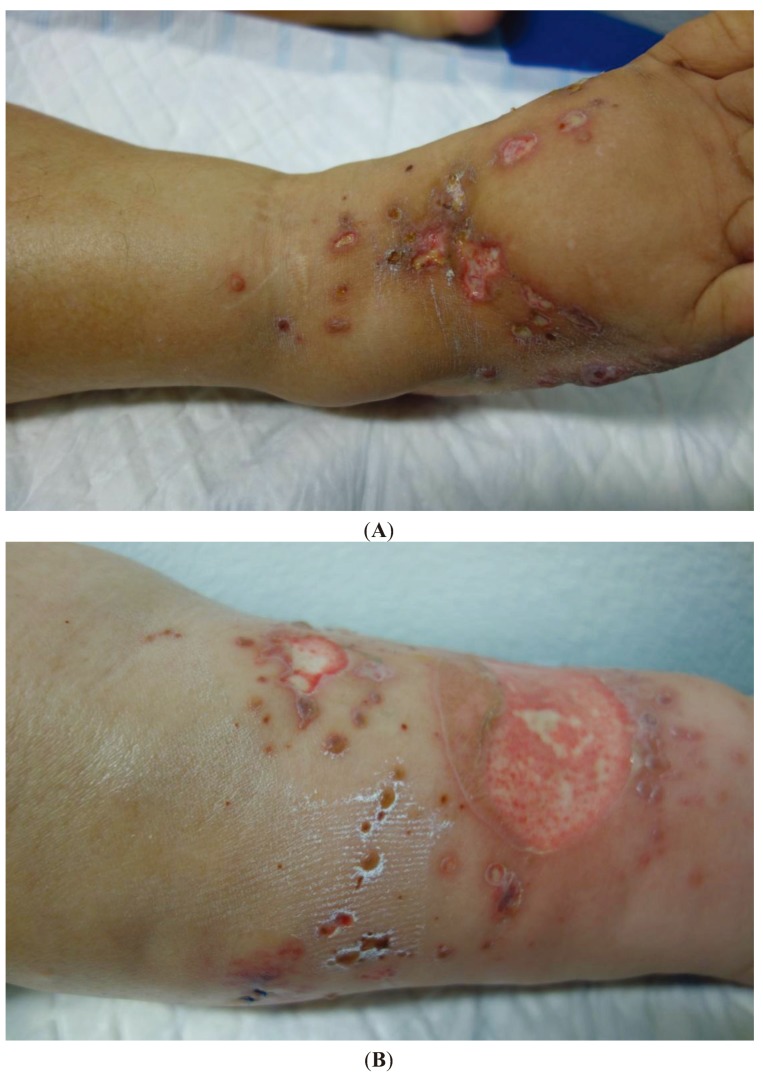
Erosions and ulcers affecting the dorsal aspect of the right foot (**A**) and the anterior, inner and outer surfaces of the left leg (**B**).

We report a 47-year-old Caucasian woman who underwent renal transplantation four months prior for end-stage renal disease due to analgesic nephropathy. Oral immunosuppressive therapy consisted of tacrolimus (5 mg *id*), mycophenolate mofetil (MMF) (500 mg *bid*) and prednisolone (15 mg *id*). The patient was referred to our department with a two-month history of bullae and painful ulcers on the lower limbs that had been previously medicated with oral cefradine for suspected bacterial infection with no improvement. She had good general condition and lacked any systemic symptoms. Physical examination showed multiple well circumscribed flaccid bullae, erosions and ulcers on an erythematous base particularly affecting the dorsal aspect of the right foot and the anterior, inner and outer surfaces of the left leg (Figure 1).

The clinical differential diagnosis included impetigo and autoimmune bullous disease. Laboratory tests revealed leukocytosis (13.3 × 10^9^ cells/L), neutrophilia 79.5% and elevated C-reactive protein of 22.6 mg/L. The culture from the ulcers exudate revealed the presence of *Pseudomonas aeruginosa*, ciprofloxacin-sensitive, and ciprofloxacin 500 mg *bid* orally was started, but no improvement was observed after 8 days of treatment.

**Figure 2:**
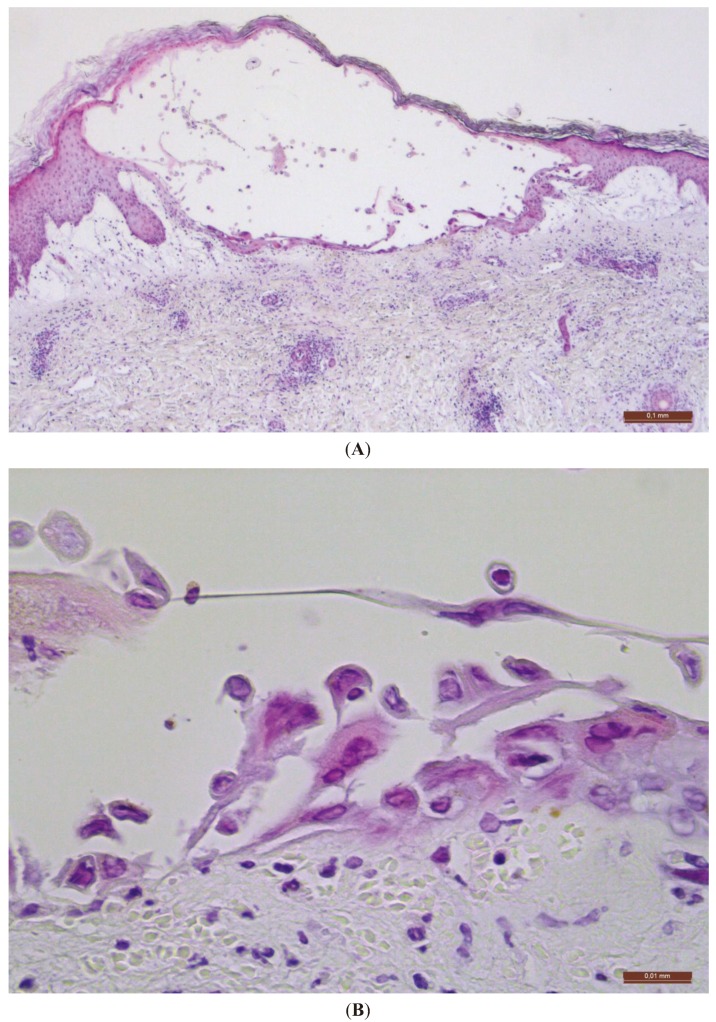
Histopathological examination. (**A**) Intraepidermal bulla (H&E, ×40). Scale bar = 0.1 mm. (**B**) Ballooning degeneration of keratinocytes and giant multinucleated (H&E, ×400). Scale bar = 0.01 mm.

Because of the atypical presentation of the lesions and the lack of clinical improvement, a skin biopsy was performed. Histopathological examination was consistent with herpetic infection (Figure 2). Serologic tests for herpes simplex virus (HSV) were IgM negative and IgG positive for HSV-1, and IgM and IgG negative for HSV-2. VZV DNA was detected by polymerase chain reaction (PCR) performed only on the biopsy sample. The patient reported a primary VZV infection in infancy and hence the diagnosis of an uncommon case of herpes zoster *duplex bilateralis* along the right L4/L5/S1 and left L4/L5/S1 dermatomes was made. The Tzanck smear was also performed in this patient, however the sample was insufficient for examination. It is an inexpensive and rapid test, with a high sensitivity and specificity for cytologic findings of herpetic infections and, therefore, a useful and practical diagnostic tool [8].

Oral valacyclovir 1 g *tid* was initiated and the department of Nephrology proposed halving the dose of MMF to 500 mg *id*, which led to a rapid response in one week and complete resolution of the lesions within three weeks with a residual hyperpigmentation. During the following six months, prophylactic therapy with oral valacyclovir 1 g daily was given, and no recurrences or symptoms of postherpetic neuralgia were reported.

Our patient had multiple bullae, erosions and ulcers on the lower limbs as a presentation of herpes zoster which represented a diagnostic challenge. Besides the atypical clinical presentation, the secondary bacterial infection may also have contributed as a confounding factor. HZ duplex is very uncommon with an incidence of less than 0.1% of all HZ cases [9], and its presentation on the lower limbs without the involvement of the trunk, face and scalp is also rare [10].

Varicela zoster virus reactivations tend to be more complicated in immunocompromised patients and can mimic other diseases, such as impetigo, autoimmune bollus diseases or vasculitis [11].

The severity of HZ infections is associated with the intensity of immunosuppression [12]. In a study performed by Mustapic Z. *et al*. [12], the introduction of MMF in an immunosuppressive protocol of renal transplant recipients resulted in a higher incidence and more severe VZV disease. Early therapy with oral acyclovir (800 mg 5 times daily) and reduction of MMF dose is a treatment option in these patients [12]. Disadvantages of acyclovir include poor bioavailability for oral administration and the resulting need for five times daily administration [13]. Oral valacyclovir (1,000 mg *tid*) and famciclovir (500 mg *tid*) can achieve higher plasma concentrations and also have a simplified dosing schedule which favor the use of this medications rather than oral acyclovir [14]. Valacyclovir shows enhanced efficacy compared with oral acyclovir, and famciclovir has a similar efficacy to acyclovir concerning the acute phase pain in immunocompromised and immunocompetent patients [13]. Brivudin is another antiviral agent that has the great advantage of requiring only once daily dosing (125 mg *id*) [15]. It can be more effective than acyclovir in reducing the duration of rash and the severity of pain, and has similar antiviral activity and tolerability comparing to famciclovir [13]. However, brivudin is not recommended for immunocompromised patients [14,15] and must not be used in combination with 5-fluorouracil or with other drugs containing 5-fluoropyrimidines [15]. In severely immunocompromised patients, intravenous acyclovir (10 mg/kg every 8 hours) remains the first-line treatment [14]. In our case, after the immunosupression was decreased with the reduction MMF dose and oral valacyclovir was initiated a complete resolution of the previous lesions was observed.

## 3. Conclusions

Decreasing cellular immunity is a predisposing factor in herpes zoster infection [16]. The introduction of more potent immunosuppressive agents including MMF may result in more infectious complications after renal transplantation [17].

In most cases, the clinical presentation of HZ allows the diagnosis to be made only by history and physical examination. However, patients who are immunosuppressed may have uncommon presentations, and require a high index of suspicion and additional diagnostic testing for proper management. HZ should be part of a differential diagnosis of cutaneous manifestations in patients who are immunocompromised.
